# A Novel, Drug Resistance-Independent, Fluorescence-Based Approach To Measure Mutation Rates in Microbial Pathogens

**DOI:** 10.1128/mBio.00120-19

**Published:** 2019-02-26

**Authors:** Erika Shor, Jessica Schuyler, David S. Perlin

**Affiliations:** a HMH Center for Discovery and Innovation, Nutley, New Jersey, USA; b Public Health Research Institute, Rutgers University-New Jersey Medical School, Newark, New Jersey, USA; Duke University; Tel Aviv University; Baylor College of Medicine

**Keywords:** *Candida glabrata*, GFP, MSH2, mutation rate

## Abstract

All evolutionary processes are underpinned by a cellular capacity to mutate DNA. To identify factors affecting mutagenesis, it is necessary to compare mutation rates between different strains and conditions. Drug resistance-based mutation reporters are used extensively to measure mutation rates, but they are suitable only when the compared strains have identical drug tolerance levels—a condition that is not satisfied under many “real-world” circumstances, e.g., when comparing mutation rates among a series of environmental or clinical isolates. Candida glabrata is a fungal pathogen that shows a high degree of genetic diversity and fast emergence of antifungal drug resistance. To enable meaningful comparisons of mutation rates among C. glabrata clinical isolates, we developed a novel fluorescence-activated cell sorting-based approach to measure the mutation rate of a chromosomally integrated *GFP* gene. We found that in Saccharomyces cerevisiae this approach recapitulated the reported mutation rate of a wild-type strain and the mutator phenotype of a *shu1Δ* mutant. In C. glabrata, the *GFP* reporter captured the mutation rate increases caused either by a genotoxic agent or by deletion of DNA mismatch repair gene *MSH2*, as well as the specific mutational signature associated with *msh2Δ*. Finally, the reporter was used to measure the mutation rates of C. glabrata clinical isolates carrying different alleles of *MSH2*. Together, these results show that fluorescence-based mutation reporters can be used to measure mutation rates in microbes under conditions of unequal drug susceptibility to reveal new insights about drivers of mutagenesis.

## Abstract (precis)

Measurements of mutation rates—i.e., how often proliferating cells acquire mutations in their DNA—are essential for understanding cellular processes that maintain genome stability. Many traditional mutation rate measurement assays are based on detecting mutations that cause resistance to a particular drug. Such assays typically work well for laboratory strains but have significant limitations when comparing clinical or environmental isolates that have various intrinsic levels of drug tolerance, which confounds the interpretation of results. Here we report the development and validation of a novel method of measuring mutation rates, which detects mutations that cause loss of fluorescence rather than acquisition of drug resistance. Using this method, we measured the mutation rates of clinical isolates of fungal pathogen Candida glabrata. This assay can be adapted to other organisms and used to compare mutation rates in contexts where unequal drug sensitivity is anticipated.

## INTRODUCTION

Acquisition of mutations underlies evolution in all contexts, and in pathogenic microbes this process can result in the emergence of dangerous drug-resistant strains. An organism’s capacity to acquire mutations is typically measured as the rate (per cell, per generation) at which a specific reporter gene accumulates mutations that result in a detectable phenotypic change. Because most genomes are extremely stable and mutation rates are typically very low, the most convenient and widely used mutation measurement assays are set up as selections, where mutation of a reporter gene confers resistance to a particular drug (1). In such an assay, mutations in the reporter gene arise at some low rate in cells proliferating in culture and are then selected by plating the cultures on drug-containing medium, which kills wild-type cells. The *CAN1* mutation assay in Saccharomyces cerevisiae is based on this principle and has been used extensively to gain insights into mechanisms controlling genome stability in yeast (2–5). Although drug resistance-based mutation assays have a number of advantages, most particularly in their relative ease and rapidity, their major limitation is that they allow direct comparisons of mutation rates only between strains that have the same level of drug sensitivity. This condition is largely satisfied when one compares the mutation rates of isogenic strains, e.g., a laboratory strain and a DNA repair mutant derived from it. However, this condition does not hold when one wishes to compare mutation rates among nonisogenic strains, e.g., a panel of clinical or environmental isolates, which may have various levels of drug tolerance. In this case, more tolerant strains are expected to survive for a longer period on selection medium, all the while continuing to produce resistance mutations, leading to an artificially high mutation rate estimate, and vice versa. Similar considerations preclude direct comparisons of mutation rates between different species, e.g., one that is highly drug susceptible versus one that is more drug tolerant, or between different growth conditions (e.g., growth in the presence of a stressor that may affect overall stress/drug tolerance). Thus, in order to rigorously measure and compare mutation rates in a way that is not restricted to a small number of laboratory strains and their derivatives, it is necessary to develop a drug resistance-independent method to measure mutation rates.

Candida glabrata is a yeast that is closely related to S. cerevisiae (6) and is also associated with the human microbiome (7). In contrast to S. cerevisiae, however, C. glabrata is an opportunistic pathogen that can cause life-threatening infections in immunocompromised individuals (8, 9). The prevalence of C. glabrata in infections has been increasing, and it is now the second most prevalent cause of invasive candidiasis in the United States and Europe (10, 11). One reason for this increase is that C. glabrata either is intrinsically resistant or acquires resistance relatively quickly to the limited number of antifungal drugs currently in clinical use (9, 12). In C. glabrata, drug resistance is predominantly caused by point mutations, either in transcription factors regulating drug efflux (13–15) or in genes encoding drug targets (16–18). Several studies have also documented the emergence of multidrug-resistant (MDR) C. glabrata infections, for which there are no treatment options and which are associated with extremely high mortality (19, 20).

Comparisons of DNA sequences (both of specific genes and of whole genomes) from different C. glabrata clinical isolates have revealed an exceedingly high level of genetic diversity, in terms of both single nucleotide polymorphisms (SNPs) and chromosomal arrangements (21–26). A multilocus sequencing typing (MLST) scheme based on SNPs at six different loci has identified over 100 distinct sequence types (STs) of C. glabrata, which cluster into seven clades (22, 25; https://pubmlst.org/cglabrata/). However, even strains within the same clade exhibit high genetic diversity (22), which, together with rapid emergence of mutations that cause drug resistance, has led to the hypothesis that C. glabrata may have a highly plastic, or mutable, genome. However, mutation rates in C. glabrata have not been measured or compared to other organisms.

In a previous study, we began to examine the role of DNA mismatch repair (MMR) in maintaining genome stability and emergence of drug resistance in C. glabrata (24). In particular, we found that different STs of C. glabrata are associated with specific SNPs in MMR gene *MSH2*, some of which result in amino acid changes and, when introduced into an *msh2Δ* reference strain on a plasmid, do not fully rescue that strain’s hypermutator phenotype. This result suggested that some C. glabrata isolates, e.g., those carrying certain variants of *MSH2*, may exhibit higher mutation rates and may therefore acquire drug resistance more rapidly. Indeed, in *Cryptococcus*, naturally occurring mutations in *MSH2* have been shown to contribute to microevolution and population diversity (59, 60). Yet, recent clinical studies have not found an association between specific *MSH2* alleles and drug resistance (27–29, 61, 62), raising the question of whether clinical isolates carrying these alleles are true mutators. To answer this question, it is necessary to measure and directly compare mutation rates between clinical isolates of C. glabrata. However, as described above, comparisons of different clinical isolates are complicated by the variation in their drug resistance profiles, some of which is due to varying activity of drug efflux pumps (14, 15, 18), which is likely to render any drug resistance-based mutation assay inapplicable.

In this study, we developed and validated a GFP-based mutation reporter that allowed us to measure mutation rates in a drug resistance-independent way. The reporter was shown to recapitulate the mutation rate and spectrum of a DNA mismatch repair mutant and detect DNA damage-induced mutagenesis in C. glabrata, recapitulate the mutation rates of wild-type and mutator strains of S. cerevisiae, and compare spontaneous mutation rates in C. glabrata and S. cerevisiae. Finally, we used this reporter to measure the mutation rates of a number of clinical isolates of C. glabrata, including those carrying a specific *MSH2* variant previously suggested to increase mutagenesis.

## RESULTS

### Developing the *GFP*-based mutation rate reporter.

To measure mutation rates in C. glabrata, at first we attempted to use traditional drug resistance-based reporters, such as *CAN1*, which has been used extensively to measure mutation rates in S. cerevisiae (2–5). In that fungus, *CAN1* cells are sensitive to the drug canavanine, whereas mutations in the *can1* gene cause canavanine resistance and can be selected on canavanine-containing plates. However, although the C. glabrata genome contains several potential *CAN1* orthologs (CAGL0J08162g and CAGL0J08184g), commonly used reference strain ATCC 2001 (also known as CBS138) was completely resistant to canavanine up to concentrations of 1 mg/ml (see Fig. S1A in the supplemental material; also data not shown), whereas the typical selection concentration in S. cerevisiae is 60 µg/ml. We also tried using 5-fluoroanthranilic acid (5-FAA), which selects for mutations in the tryptophan biosynthetic pathway (30). Although ATCC 2001 and many clinical isolates of C. glabrata were sensitive to 5-FAA, we discovered that this sensitivity widely varied among different strains (Fig. S1B). Although this variation was not entirely surprising, as different clinical isolates are well known to show different levels of antifungal drug resistance, which is at least in part due to the activity of drug efflux pumps, it also eliminated the possibility of using 5-FAA—or likely any other drug resistance-based approach—to measuring mutation rates in C. glabrata clinical isolates.

10.1128/mBio.00120-19.1 FIG S1 Drug resistance-based mutation reporters are not suitable to measure mutation rates in C. glabrata. (A) C. glabrata is highly resistant to canavanine. (B) Different clinical isolates (numbers 1 through 9) show various levels of sensitivity to 5-FAA, a drug that can be used to select for mutations in the tryptophan biosynthesis pathway. Download FIG S1, PDF file, 0.9 MB. Copyright © 2019 Shor et al. 2019 Shor et al. This content is distributed under the terms of the Creative Commons Attribution 4.0 International license.

**FIG 1:**
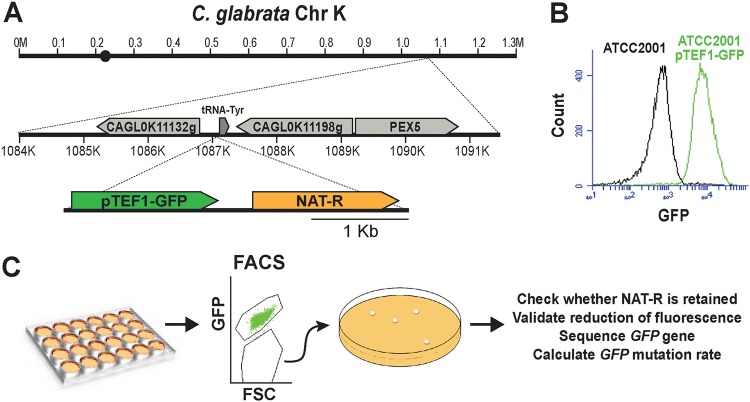
Description of the fluorescence-based mutation reporter. (A) A cassette containing *pTEF1-*driven *yEGFP* linked to the gene encoding nourseothricin resistance (*NAT*) under its own promoter was integrated into the indicated locus on chromosome K in C. glabrata laboratory strain ATCC 2001. (B) The resulting strain became constitutively fluorescent. (C) Schematic describing the fluctuation assay used to measure the mutation rate of *yEGFP*.

To enable measurements of mutation rates in a way that was independent of drug resistance, we chose a fluorescence-based approach. We created a cassette where the gene encoding yeast enhanced green fluorescent protein (*yEGFP*) was driven by the strong constitutive promoter *pTEF1* of S. cerevisiae (31), which was also previously shown to strongly induce gene expression in C. glabrata (32) (Fig. 1A). In order to facilitate the chromosomal insertion and subsequent tracking of this construct, the cassette also contained the gene conferring nourseothricin resistance (*NAT*) driven by its own promoter (Fig. 1A). This cassette was inserted into the right arm of C. glabrata chromosome K between two uncharacterized ORFs (Fig. 1A) and validated by sequencing. The resulting strain was constitutively and strongly fluorescent (Fig. 1B) and was used to measure mutation rates of *yEGFP* using fluorescence-based cell sorting (FACS) in fluctuation experiments as described below (Fig. 1C; Fig. S2).

10.1128/mBio.00120-19.2 FIG S2 Data from a representative FACS experiment. Cells were first gated based on SSC-W versus SSC-H to identify singlets, then based on PI staining to identify viable cells, and finally based on GFP fluorescence. Although there is a range of fluorescence in both the nonfluorescent strain (bottom left) and the fluorescent strain containing the reporter (bottom right), the two cell populations are well separated in terms of fluorescence intensity, allowing the sorting of rare nonfluorescent cells (bottom right, negative gate) from the fluorescent population. Download FIG S2, PDF file, 1.2 MB. Copyright © 2019 Shor et al. 2019 Shor et al. This content is distributed under the terms of the Creative Commons Attribution 4.0 International license.

Briefly, in a typical experiment, a starter YPD culture was diluted into multiple (e.g., 8 to 12) parallel YPD cultures in a 96-well plate to a starting density of a few cells per well and incubated at 37°C overnight. The following morning, each culture, in its entirety, was diluted severalfold in YPD to ensure that cells collected for FACS analysis several hours later were in log phase, which was found to be necessary to achieve maximum expression of GFP and the optimal resolution between GFP-positive and GFP-negative populations. The cells were then collected by filtration, resuspended in PBS, and analyzed by FACS. Prior to FACS analysis, propidium iodide (PI) was added to each sample to gate out the inviable, PI-positive cell subset (Fig. S2). Although GFP levels of fluorescent cultures varied slightly between experiments (e.g., between the same strain analyzed on different days and between different strains), the overall fluorescence levels of GFP^+^ cells were always significantly higher than those of GFP^−^ cells (Fig. S3), allowing for efficient sorting of GFP^−^ cells from GFP^+^ populations.

10.1128/mBio.00120-19.3 FIG S3 GFP-positive populations are always clearly separated in terms of fluorescence levels from GFP-negative populations. Although small degrees of variation in fluorescence were observed between different strains with the integrated GFP construct, as well as between the same strain analyzed on different days, GFP-positive populations always fluoresced much more strongly than GFP-negative populations, allowing for efficient sorting of GFP-negative cells from GFP-positive cultures. Download FIG S3, PDF file, 1.7 MB. Copyright © 2019 Shor et al. 2019 Shor et al. This content is distributed under the terms of the Creative Commons Attribution 4.0 International license.

The number of cells per culture (*n*) was optimized by varying the final volume of the cultures. For strains with lower mutation rates, such as the reference C. glabrata strain ATCC 2001, the optimal *n* was found to be ≥3 × 10^6^ cells/well, whereas for cells with elevated mutation rates (such as *msh2Δ*), 1 × 10^6^ cells/well was sufficient to obtain multiple cultures with mutants. Each culture, in its entirety, was analyzed by FACS, and GFP-negative cells were collected, immediately plated onto YPD plates, and allowed to form colonies. These colonies were then validated for (i) the presence of the NAT cassette (by replica plating onto nourseothricin medium) and (ii) reduction of GFP fluorescence (by flow cytometry). Next, *yEGFP* was sequenced in NAT^+^ GFP^−^ colonies to identify the mutations responsible for reduced fluorescence. For every strain, 200 GFP^+^ cells were also collected by FACS and plated on YPD to calculate plating efficiency. Finally, mutation rates and 95% confidence intervals were calculated using the MSS maximum likelihood method (1, 33) based on *n* (the number of processed cells × PI-negative fraction × plating efficiency) and the number of NAT^+^ GFP^−^ mutants in every culture (*r*). Importantly, we found that every NAT^+^ GFP^−^ colony in which GFP was sequenced contained a single mutation in the *yEGFP* ORF or, in a few cases, the *pTEF1* promoter (see below), indicating that loss of fluorescence is virtually always caused by mutations in *yEGFP* and that therefore this mutation assay is highly specific to a single locus.

### Mutations in *GFP* do not affect cellular fitness.

**FIG 2:**
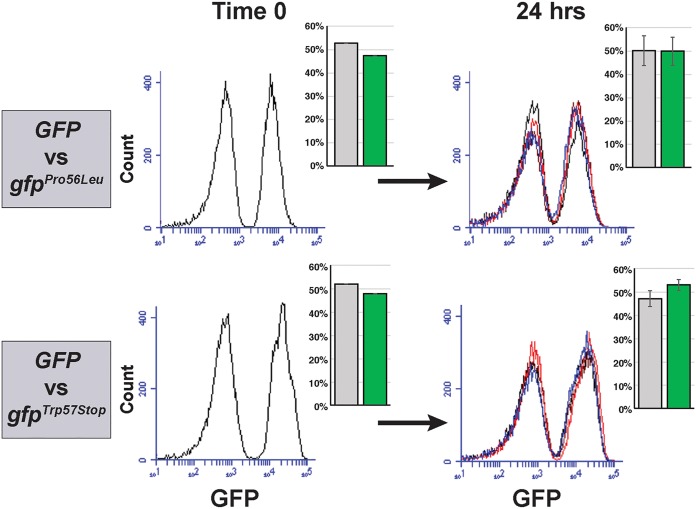
Mutation of *yEGFP* does not impact fitness relative to the fluorescent parent strain. Two different *yEGFP* mutants were each cocultured together with the fluorescent parent strain for 24 h. The relative proportions of the strains carrying wild-type and mutant *yEGFP* genes, quantified and shown as bar graphs, remained constant over the course of the experiment, indicating no difference in relative fitness.

A key condition that has to be satisfied by any mutation rate measurement assay is that mutations in the reporter gene should not affect the fitness of the strain, either positively or negatively, as this would result in overestimating or underestimating the mutation rate, respectively (1). To check that this condition is fulfilled in the GFP-based mutation reporter, we isolated two different loss-of-function mutations in *yEGFP* using FACS and measured their fitness compared to the parent strain over 24 h of growth in YPD, which is the duration of a typical mutation rate experiment. Each strain was mixed 1:1 with the parent strain, producing a coculture where approximately half the population was fluorescent and half was not (Fig. 2, Time 0). Both cocultures were diluted into multiple wells to several hundred cells per well and grown for 24 h at 37°C, mimicking a typical fluctuation experiment. After 24 h, fluorescence measurements showed that the proportions of fluorescent and nonfluorescent cells in the cultures had not significantly changed (Fig. 2, 24 h), indicating that mutations in *yEGFP* did not affect fitness relative to the parental GFP-positive strain.

### *GFP-*based mutation reporter recapitulates mutation rates in Saccharomyces cerevisiae.

**FIG 3:**
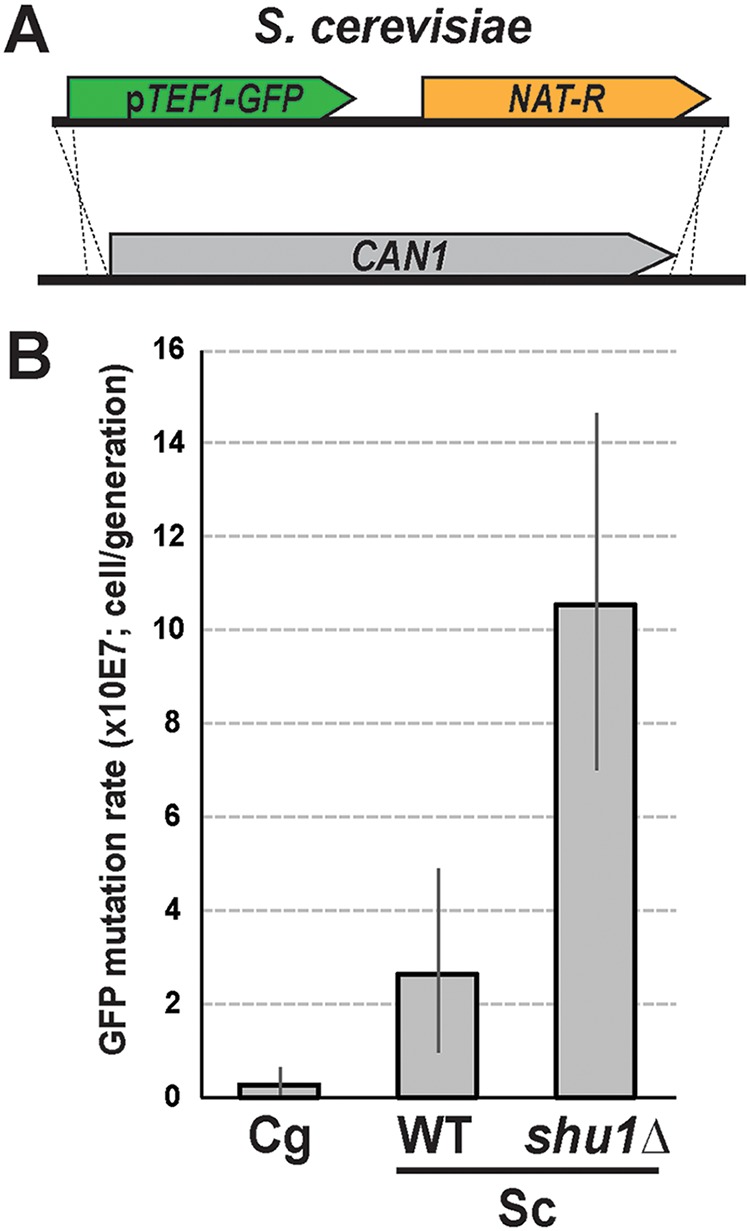
Fluorescence-based mutation reporter recapitulates the mutation rate increase of an S. cerevisiae mutant. (A) The *yEGFP-NAT* cassette was integrated at the *CAN1* locus of S. cerevisiae using homology-based targeting. (B) The assay detected the mutator phenotype of the *shu1Δ* mutant, previously shown to have an elevated *CAN1* mutation rate (34). The assay also showed that spontaneous forward mutation in C. glabrata during standard laboratory growth in YPD is not higher than that in S. cerevisiae. Sc, S. cerevisiae; Cg, C. glabrata. Error bars, 95% confidence intervals.

In addition to inserting the GFP cassette into the C. glabrata genome, we also inserted it at the *CAN1* locus in S. cerevisiae (Fig. 3A) in order to test whether this reporter would recapitulate the previously determined mutation rates of two S. cerevisiae strains: a wild-type strain of the W303 background and an isogenic mutant carrying a deletion of the *SHU1* gene. *SHU1* functions in promoting error-free DNA repair by homologous recombination, and its loss was shown to increase *CAN1* mutation rate by approximately 8.5-fold in one study (34) and by 4-fold in another study (35). The FACS-based assay described above (Fig. 1C) was used to measure the mutation rates of *yEGFP* in wild-ype and *shu1Δ* strains. The assay recapitulated the increase in mutation rate in the *shu1Δ* mutant relative to the wild-type strain (4-fold; Fig. 3B). Furthermore, the absolute mutation rate obtained for the wild-type strain was ∼2.7 per 10^7^ cells per generation, which agrees well with typical mutation rates obtained in S. cerevisiae (2, 5, 34).

Finally, using the fluorescence-based mutation reporter, we were able to directly compare spontaneous mutation rates at the C. glabrata locus carrying the cassette (Fig. 1A) and the *CAN1* locus of S. cerevisiae. Interestingly, we found that the mutation rate was 9-fold higher at the S. cerevisiae *CAN1* locus than at the analyzed locus in C. glabrata (Fig. 3B), suggesting that, at least during unperturbed growth in YPD, the examined strain of C. glabrata (ATCC 2001) does not behave as a spontaneous hypermutator.

### *GFP*-based mutation reporter captures the *msh2Δ* mutator phenotype and mutation spectrum.

**FIG 4:**
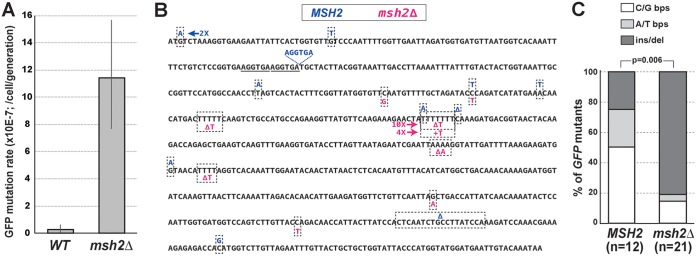
Fluorescence-based mutation reporter captures the mutator phenotype and mutational signature of the *msh2Δ* DNA mismatch repair mutant in C. glabrata. (A) Using CRISPR, the *yEGFP-NAT* cassette was integrated into the same genetic locus in an ATCC 2001-derived strain carrying a deletion of *MSH2*. The mutation rate of *yEGFP* was measured and found to be increased 40-fold by *msh2Δ* relative to the strain carrying wild-type *MSH2*. Error bars, 95% confidence intervals. (B and C) Sequencing of *yEGFP* in nonfluorescent mutants isolated by FACS from *MSH2* and *msh2Δ* strains identified a number of mutations throughout the *yEGFP* ORF. Whereas mutations in the *MSH2* strain were mostly base pair substitutions, the majority of mutations in the *msh2Δ* strain were single nucleotide frameshifts (insertions or deletions) in mononucleotide runs, recapitulating the mutational signature of *msh2Δ* in S. cerevisiae (37, 38, 41). bps, base pair substitution; ins/del, insertion or deletion.

To further validate the GFP-based mutation reporter in C. glabrata, we used CRISPR to insert the cassette into the same chromosomal location (Fig. 1A) in a C. glabrata strain derived from ATCC 2001 but carrying a deletion of DNA mismatch repair (MMR) gene *MSH2* (24). *MSH2* is the C. glabrata homolog of MutSβ, whose loss has been shown to result in a strong mutator phenotype in all organisms where it has been examined, including C. glabrata (24, 36). Both *MSH2* and *msh2Δ* strains were used in fluctuation experiments to measure their mutation rates as described above (Fig. 1C and Fig. S2). We found that, as expected, *msh2Δ* resulted in a strong mutator phenotype, increasing the rate of mutation of *yEGFP* by 40-fold (Fig. 4A), which is very similar to its effect on mutation rates in S. cerevisiae, where the *msh2Δ* effect on *CAN1* mutation rate ranges from a 16- to a 40-fold increase, depending on the study (37–40). Furthermore, sequencing GFP^−^ colonies revealed that the *msh2Δ* strain produced a very different spectrum of mutations in *yEGFP* from the *MSH2* strain (Fig. 4B and C). Mutations in the wild-type (*MSH2*) strain were largely comprised of base pair substitutions (bps), whereas the majority of mutations in the *msh2Δ* mutant were due to single nucleotide deletions or insertions in mononucleotide repeats (e.g., AAAA or TTTT), with the strongest mutation “hot spot” at a run of seven T’s (Fig. 4B). This mutation spectrum recapitulates that of the *msh2Δ* strain in S. cerevisiae and is thought to be due to DNA polymerase “slippage” errors at mononucleotide repeats, which are normally repaired by MMR (37, 38, 41). Thus, the GFP-based mutation reporter was able to accurately capture both the increase in mutation rate and the change in mutation spectrum of the *msh2Δ* mutant.

### *GFP-*based mutation reporter detects DNA-damage induced mutagenesis.

**FIG 5:**
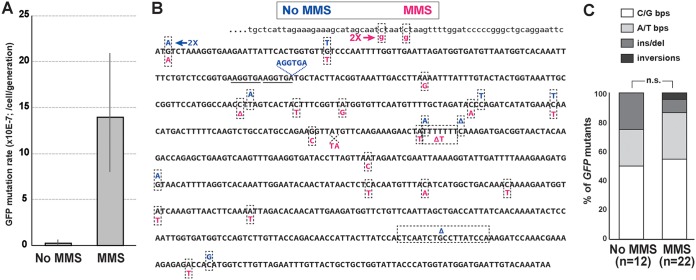
Fluorescence-based mutation reporter captures the elevated mutation rate in C. glabrata caused by growth in the presence of genotoxic agent MMS. (A) C. glabrata strain ATCC 2001 carrying the fluorescent reporter was cultured in the presence of 0.01% MMS, and its mutation rate was measured as described in the text and in Fig. 1. Error bars, 95% confidence intervals. (B and C) Sequencing of *yEGFP* in nonfluorescent mutants that formed in the presence of MMS showed a mutation spectrum similar to that produced in the absence of the drug. Several mutations generated during growth in the presence of MMS were in the promoter region (lowercase letters, top row). bps, base pair substitution; ins/del, insertion or deletion.

To investigate whether the GFP-based mutation reporter would capture DNA damage-induced mutagenesis, we performed the fluctuation assay on the ATCC 2001 strain grown in the presence of 0.01% methyl methanesulfonate (MMS), an alkylating agent. Indeed, we detected a 48-fold increase in *yEGFP* mutation rate in cells cultured in the presence of MMS (Fig. 5A). Sequencing mutations in GFP^−^ colonies recovered after growth in MMS showed that the spectrum of mutations did not change significantly from that in cells grown in the absence of MMS (Fig. 5B and C). Thus, MMS caused an overall increase in mutagenesis of *yEGFP*, as expected, but apparently did not significantly change the cellular pathways by which these mutations were generated. This was consistent with a previous report, where the MMS-induced mutation spectrum in yeast was similar to the spontaneous mutation spectrum (42).

### Mutation rates of C. glabrata clinical isolates.

**FIG 6:**
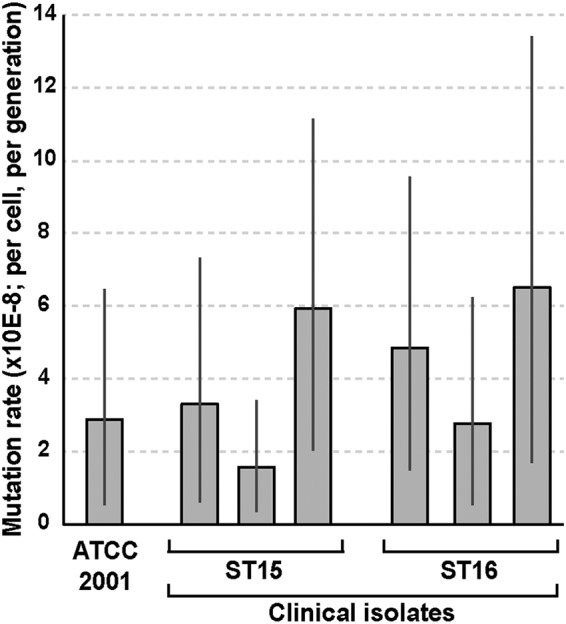
Fluorescence-based mutation reporter reveals similar mutation rates in a panel of clinical C. glabrata isolates. Using CRISPR, the *yEGFP-NAT* cassette was integrated into the same genomic locus in six clinical isolates of C. glabrata. Three of these isolates, like ATCC 2001, belonged to ST15 and therefore carried the same *MSH2* allele, whereas the other three belonged to ST16, which carries the *MSH2^E231G/L269F^* variant (23). Mutation rates of *yEGFP* were measured as described above and found to be similar among all examined isolates irrespective of *MSH2* sequence. Error bars, 95% confidence intervals.

We used the GFP-based mutation assay to measure mutation rates of six clinical C. glabrata isolates: three that, like ATCC 2001, belonged to sequence type (ST) 15 and carried the corresponding *MSH2* sequence and three belonging to ST16 and carrying the variant *MSH2^E231G/L269F^* (23, 24). For every clinical isolate, the reporter cassette was integrated into the same chromosomal locus using CRISPR and validated by flow cytometry and DNA sequencing, and the mutation rate was measured using FACS as described above. Interestingly, we found that none of the clinical isolates, including those carrying *MSH2^E231G/L269F^*, had elevated mutation rates relative to the reference strain ATCC 2001 (Fig. 6). Thus, even though our previous examination indicated that *MSH2^E231G/L269F^* did not fully rescue the mutator phenotype of the *msh2Δ* mutant in ATCC 2001 (24), a direct, rigorous assessment of the mutation rate of the clinical isolates showed that under standard laboratory conditions this variant, in its native genomic context, is not associated with an elevated spontaneous forward mutation rate.

## DISCUSSION

The goal of this study was to develop, validate, and use a new method for measuring mutation rates in a way that did not rely on drug resistance-based reporters. To this end, we designed a FACS-based scheme to capture and quantify loss-of-function mutations in the gene encoding GFP and used it in fluctuation experiments to measure mutation rates in C. glabrata and S. cerevisiae. We found that this fluorescence-based mutation reporter recapitulated the previously reported mutator phenotype of the S. cerevisiae *shu1Δ* mutant and captured the expected increase in mutation rates due to loss of *MSH2* or treatment with a genotoxic agent in C. glabrata. This reporter also accurately captured the mutational spectrum (a predominance of single nucleotide insertions or deletions in homopolymeric runs) associated with loss of *MSH2*. Finally, the reporter was used to measure the mutation rates of several clinical C. glabrata isolates, including those carrying the *MSH2^E231G/L269F^* variant previously suggested to contribute to increased mutagenesis (24), and showed that all clinical isolates examined had very similar spontaneous mutation rates.

Our mutation assay showed that under standard laboratory growth conditions (YPD, 37°C), C. glabrata clinical isolates carrying the *MSH2^E231G/L269F^* variant do not show elevated spontaneous mutation rates relative to ATCC 2001 or to clinical isolates carrying the “wild-type” version of *MSH2* (i.e., one identical to that in ATCC 2001). This is consistent with several recent clinical studies that did not see an association between *MSH2* genotype and prevalence of drug resistance in C. glabrata (27–29, 61, 62). However, in our previous study, we found that several *MSH2* variants, including *MSH2^E231G/L269F^*, when introduced on a plasmid into an ATCC 2001-derived *msh2Δ* mutant, did not fully rescue that strain’s elevated mutation rate (24). There are several non-mutually exclusive possibilities that can reconcile previous data with this present study. First, it is possible that, although the plasmids were maintained by selection, a subpopulation of the culture had lost the plasmid and therefore lacked any copy of *MSH2*. Second, it is possible that the *MSH2* variants carried on a plasmid were present in more than one copy, which, depending on the nature of the mutation, might either help restore function (for a partial loss-of-function mutation) or further exacerbate the associated defects (for a dominant negative mutation). Finally, it is important to consider that in the clinical isolates studied here, the *MSH2^E231G/L269F^* variant is present in its normal genomic context, which is that of ST16 (23). ST16 is separated from ATCC 2001 (ST15) by hundreds to thousands of SNPs throughout the genome, including SNPs in genes that encode protein partners of Msh2, such as Msh3 and Msh6 (36). Thus, it is possible that each *MSH2* variant has evolved in concert with its partner genes in a way that maintains efficient MMR and low mutation rates. In this scenario, moving a particular variant to a different genomic context would force it to form suboptimal partnerships with noncognate interacting proteins, resulting in less efficient MMR and an increased spontaneous mutation rate. Consistent with this hypothesis, our analyses of *MSH3* and *MSH6* sequences have revealed that each of these genes has multiple SNPs between ST15 and ST16 strains, including five SNPs in each gene that result in amino acid changes (the ST15 and ST16 whole-genome sequences have been generated by us for a different project [unpublished data]).

The fluorescence-based mutation assay developed in our study has specific advantages and specific limitations that need to be considered when deciding whether to use it or another assay to measure mutation rates in a given experimental system. First, similar to other mutation assays based on a loss of function of a reporter, only mutations that affect GFP function, i.e., its ability to fluoresce in the detectable range, can be identified. Thus, if it is desirable to calculate true mutation rates independently of whether a mutation is expressed, it is more appropriate to use the recently developed whole-genome sequencing (WGS) approaches (43–45). Although this is a very powerful technique, it requires WGS of multiple isolates per strain and is therefore still considerably more expensive and computationally heavy than our method, which requires no computational expertise. One unique hurdle of our assay not shared by other methods is that it requires access to a FACS instrument that can sort millions of cells in minutes; otherwise, the time frame of a single experiment becomes unfeasibly long. Once the sorting is completed, however, the rest of the steps require a standard flow cytometer (or fluorescence microscope) and standard laboratory techniques. As discussed above, this assay is going to be more informative than drug resistance-based assays when the strains under comparison have different drug tolerance profiles, as can be expected for environmental/clinical isolates or different species or when one wishes to measure the mutation rates of strains exposed to different types of environmental stress (e.g., antimicrobial drugs).

We validated our mutation assay by analyzing strains with elevated mutation rates (e.g., *shu1Δ* in S. cerevisiae and *msh2Δ* in C. glabrata). It should also be possible to use this assay to identify antimutators—i.e., genes whose loss reduces mutagenesis—which can be extremely informative for identifying cellular pathways that promote mutagenesis and genetic instability (46–48). However, this would require sorting cultures with significantly larger numbers of cells and would therefore take longer, with the sorting time required negatively correlating with the mutation rate of the strain. One potential improvement over the current methodology that would reduce the required time and labor is developing a fluorescence-based assay that uses flow cytometry to count the nonfluorescent cells but skips the sorting and plating steps. In our current setup, this was not possible because the number of GFP-negative cells recorded by the FACS instrument was typically much greater than, and did not correlate with, the number of colonies that grew from the sorted cells. In other words, despite our use of propidium iodide (PI) to gate out membrane-permeable cells, a large subset of PI-negative GFP-negative cells were inviable/nonculturable. Perhaps, with further optimization—e.g., using other fluorescent markers that can be used in conjunction with live/dead dyes—it will be possible to accurately record the number of nonfluorescent live cells using flow cytometry only, without the need for sorting and plating.

We have used the fluorescence-based assay to measure forward mutation rates; however, because the reporter cassette contains two genes (*yEGFP* and *NAT*), it can be adapted to measure large deletions by looking for simultaneous loss of both *yEGFP* and *NAT*, similar to the *CAN1-URA3* loss assay developed in S. cerevisiae (49). Such an assay would be extremely useful in C. glabrata and other fungal pathogens characterized by frequent genomic rearrangements (26, 50, 51). In the current genomic location of the cassette, we did not identify any simultaneous deletions of *yEGFP* and *NAT* among the >170 analyzed C. glabrata cultures, including those containing genotoxic agent MMS, indicating that the spontaneous rate of deletions at this locus is extremely low. In future studies, the *yEGFP-NAT* cassette will be integrated at genomic loci more likely to undergo rearrangements, such as subtelomeric loci containing multiple and variable numbers of genes from the adhesin family (52, 53) and ribosomal DNA (rDNA) (54).

The high degree of genetic diversity in C. glabrata populations and the fast emergence of drug resistance both indicate that at least under some conditions, C. glabrata is able to rapidly mutate and diversify its genome. Our present study indicates that these conditions do not include unperturbed growth in YPD, suggesting that C. glabrata may be subject to stress-induced mutagenesis. Indeed, previously, stress-induced mutator phenotypes have been identified in a majority of natural isolates of Escherichia coli, whereas only 5% were shown to act as constitutive mutators (55). Future studies will examine whether mutagenesis is affected by stress conditions, including those encountered by C. glabrata in the host, such as oxidative stress and exposure to antifungal drugs. The fluorescence-based mutation assay is particularly well suited to address such questions because its outcome is independent of whether the strain’s sensitivity to stress is altered by an exogenous treatment (e.g., by an antifungal drug). This assay can also be adapted and used to address questions regarding mechanisms driving mutagenesis in other clinically relevant microbes, including bacterial pathogens and haploid pathogenic fungi, such as Candida auris, Cryptococcus neoformans, and Candida lusitaniae, where emergence of drug resistance poses a serious public health threat.

## MATERIALS AND METHODS

### Construction of the GFP reporter cassette and creating fluorescent strains for fluctuation analyses.

The S. cerevisiae *TEF1* promoter was amplified using primers CACACCAGAGCTCCAAAATGTTTCTACTCC and CCATTTTGGATCCAAAACTTAGATTAGATTGC and subcloned into the BamHI-SacI sites of pYC54 (56), placing it directly upstream of the YFP ORF. Next, the YFP ORF was replaced by that of yEGFP as follows. *yEGFP* was amplified from pGRB2.3 (57) using primers ACTAGTGGATCCCCCGGGCTGCAGGAATTCATG and CGAATTGGCTAGCTTTACCTCTATATCGTGTTCG and subcloned into the plasmid using BamHI-NheI sites. The final plasmid contained the pTEF1-yEGFP-NAT cassette (Fig. 1A). This cassette was amplified from the plasmid using primers CCCCTCGAGGACGAAGTTCC and TGTAATACGACTCACTATAGGGCG and transformed into C. glabrata strain ATCC 2001 using nourseothricin resistance as selection.

Because there were no targeting homology sequences on the cassette, it integrated randomly into the C. glabrata genome. Several independent, constitutively fluorescent transformants were chosen and submitted for whole-genome sequencing at the New Jersey Medical School Molecular Resource Facility using the NextSeq (Illumina, San Diego, CA) platform. Libraries were prepared with the Nextera XT kit (Illumina, San Diego, CA) to produce paired-end reads of 150 bp for an approximate minimum coverage of 100×. Data analysis was performed using CLC Genomics Workbench (Qiagen, Hilden, Germany). Each transformant was found to contain a single integration of the cassette. The transformant carrying the cassette integrated between uncharacterized ORFs CAGL0K11132g and CAGL0K11198g on Chr K (strain ESCg36), as shown in Fig. 1A, was chosen for further analysis.

In all other C. glabrata strains reported in this study, the cassette was integrated at the same genomic locus as in ESCg36 using CRISPR-mediated targeted integration. The cassette was amplified from ESCg36 using primers GCAGTCTTTCTTGATCCACATATC and CACAGAATTGGTAGGACGGG, which produced approximately 500-nt 5′ and 3′ homology each to the desired integration locus. CRISPR was performed as in reference 58, except that cells were made competent for electroporation using the Frozen-EZ yeast transformation kit (Zymo Research) according to the manufacturer’s instructions.

To replace the *CAN1* ORF of S. cerevisiae with the *yEGFP-NAT* cassette, the cassette was amplified using primers AAAAGGCATAGCAATGACAAATTCAAAAGAAGACGCCGACATAGAGGACCAGTGAATTGTAATACGACTC and AGGTAATAAAACGTCATATCTATGCTACAACATTCCAAAATTTGTCCTGGTACCGGGCCCCCCCTCGAG, which introduced 48-nt regions of homology directly 5′ upstream and 3′ downstream of the *CAN1* ORF. This PCR product was transformed into S. cerevisiae strains W4069-4C (wild-type W303 *MAT***a**) and W4220-15A (*shu1Δ*::*HIS3 MAT***a**) using the Frozen-EZ yeast transformation kit (Zymo Research) according to the manufacturer’s instructions. Transformants were selected on nourseothricin plates as described above and validated by sequencing of the *CAN1* locus and by flow cytometry to verify the acquisition of green fluorescence.

All primers were ordered from Integrated DNA Technologies, and all Sanger sequencing of the above-described constructs was done by Genewiz.

### Measuring *yEGFP* mutation rates using fluctuation analysis and FACS.

A starter YPD culture of the strain whose mutation rate was being measured was diluted into multiple (e.g., 8 to 12) parallel YPD cultures in a 96-well plate to a starting density of several hundred cells per well and incubated overnight at 37°C (C. glabrata) or 30°C (S. cerevisiae). The following morning, each culture was diluted severalfold in YPD and grown at the same temperature to ensure that when the cells were collected for FACS several hours later, they were in log phase, which was found to be necessary to achieve maximum expression of GFP and the best resolution between GFP-positive and GFP-negative populations. The cells were then collected by filtration using 0.45-µm mixed cellulose ester membrane filters (Millipore), resuspended in PBS, and sorted using the BD FACSAria II (BD Biosciences) at the New Jersey Medical School Flow Cytometry and Immunology Core Laboratory. Ten to 15 min before sorting, each sample was stained with 10 µg/ml propidium iodide (PI; ThermoFisher) to identify and gate out inviable cells. FSC and SSC parameters were set on log scale. Cells were gated for singlets (SSC-W versus SSC-H) followed by live gating on PI negative. Finally, cells were sorted based on GFP expression. GFP-negative cells from each entire culture were collected into microcentrifuge tubes containing 250 µl YPD and then plated immediately onto YPD agar plates. Two hundred GFP-positive cells were also sorted and plated to calculate plating efficiency. The plates were incubated at 37°C (C. glabrata) or 30°C (S. cerevisiae) to allow the sorted cells to form colonies.

The resulting colonies were checked for the presence of the *NAT* marker by replica plating or patching onto plates containing 100 µg/ml nourseothricin (Jena Bioscience) and then checked for the level of green fluorescence using a BD Accuri C6 flow cytometer (BD Biosciences). Mutation rates and 95% confidence intervals were calculated using the MSS maximum likelihood method (1, 33) based on the number of NAT^+^ GFP^−^ mutants in every culture (*r*) and the average number of viable cells per culture (*n*). To identify the mutations responsible for loss of GFP fluorescence, the *yEGFP* ORF and promoter were sequenced using primers CTCTTTCGATGACCTCCCATTG and TGTAATACGACTCACTATAGGGCG, respectively.
