# Supplementary figures and images for: A Novel, Drug Resistance-Independent, Fluorescence-Based Approach To Measure Mutation Rates in Microbial Pathogens
Source: mBio. 2019 Feb 26;10(1):e00120-19. doi: 10.1128/mBio.00120-19 (PMC6391916; doi:10.1128/mBio.00120-19)

Supplementary figure 1.

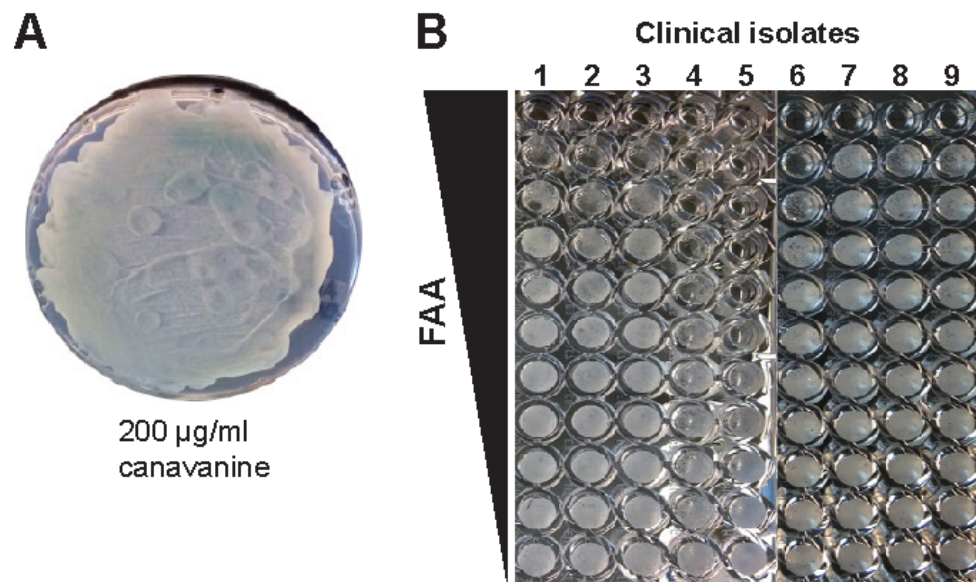

Supplement: FIG S1 [file mBio.00120-19-sf001.pdf]

Supplementary figure 2.

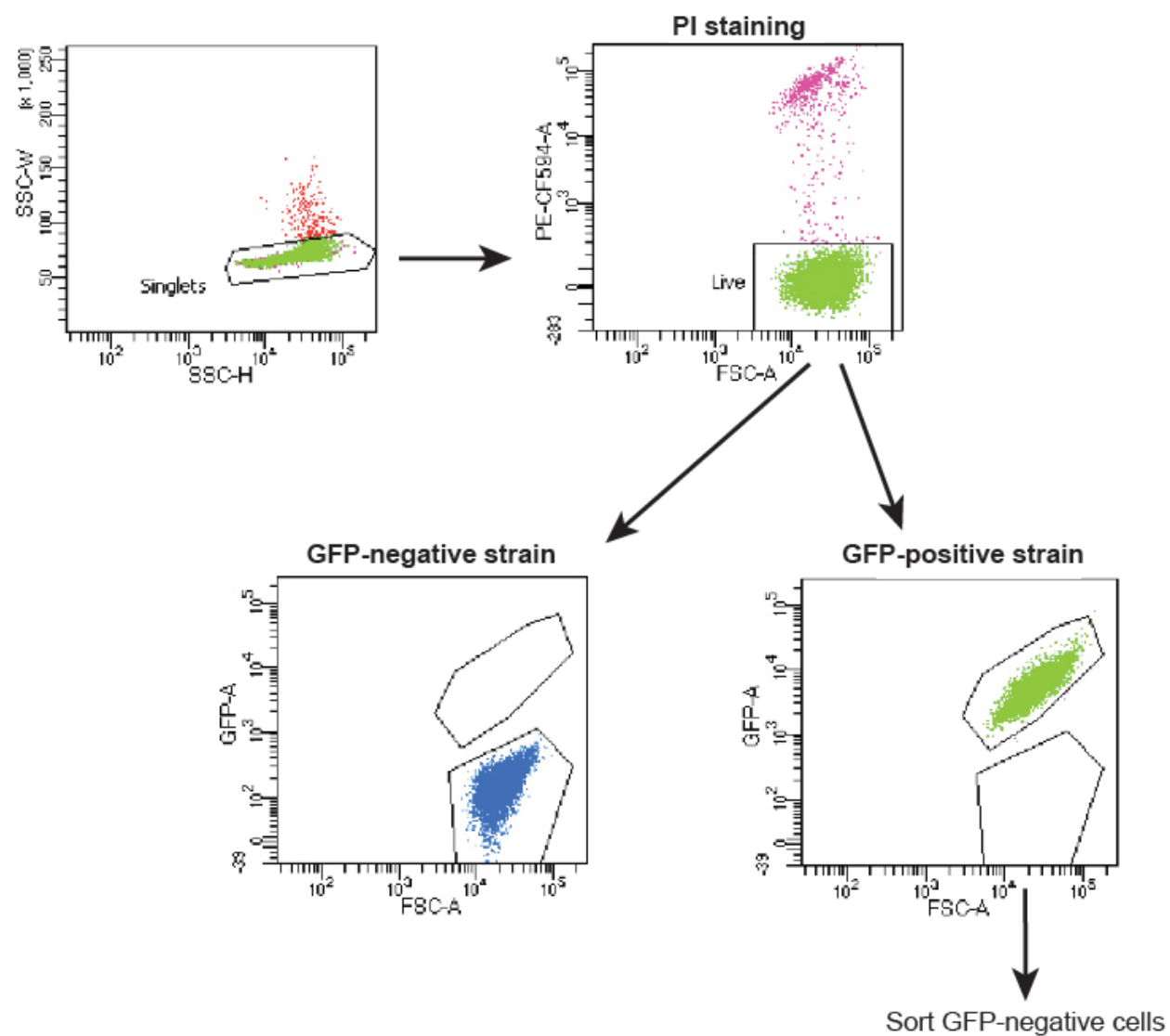

Supplement: FIG S2 [file mBio.00120-19-sf002.pdf]

Supplementary figure 3.

ATCC2001

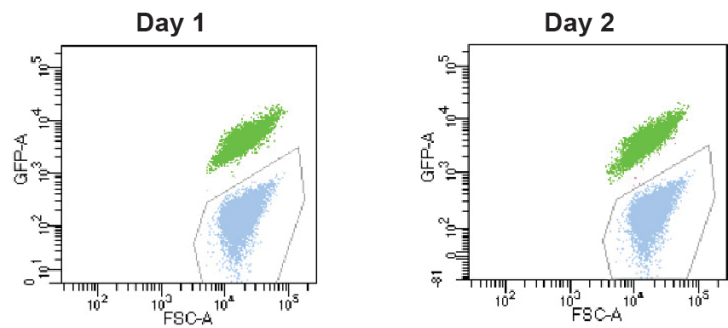

Clinical isolate 1

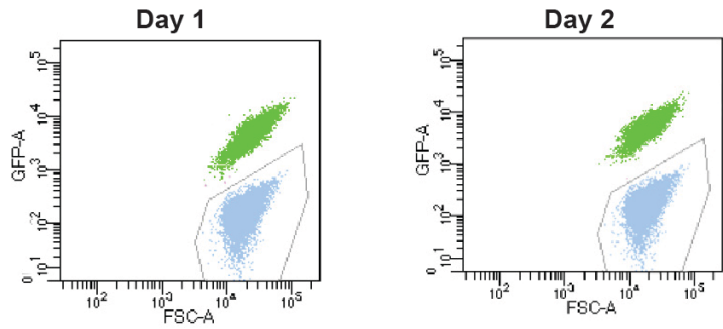

Clinical isolate 2

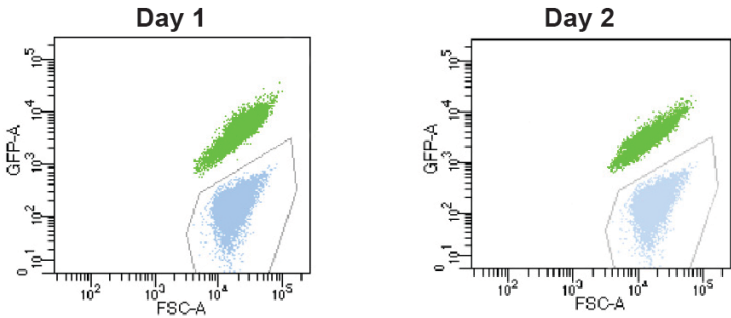

Supplement: FIG S3 [file mBio.00120-19-sf003.pdf]
